# The Effects of Health Literacy, Social Support, and Health-Promoting Behaviors on Metabolic Syndrome Among Middle-Aged and Older Women Living in Rural Areas of Republic of Korea

**DOI:** 10.3390/healthcare13243279

**Published:** 2025-12-13

**Authors:** Eun-Kyung Lee, Yong-Sook Eo

**Affiliations:** College of Nursing, Dongguk University WISE, Gyeongju 38066, Republic of Korea; ruddl5238@naver.com

**Keywords:** health literacy, social support, health promoting behavior, metabolic syndrome

## Abstract

**Highlights:**

**What are the main findings?**

- Obesity, menopausal status, perceived health, health literacy, and health-promoting behaviors are significant predictors of metabolic syndrome among middle-aged and older women in rural Korea.
- Higher levels of health literacy and engagement in health-promoting behaviors are associated with a lower risk of metabolic syndrome.

**What are the implications of the main findings?**

- Enhancing health literacy and fostering proactive health responsibility behaviors can reduce metabolic syndrome among rural women.
- Community-based tailored health education and health literacy programs are es-sential for improving health equity and preventing metabolic disorders among rural women.

**Abstract:**

Background/Objectives: This study examined how health literacy, social support, and health-promoting behaviors influence the prevalence of metabolic syndrome among middle-aged and older women living in rural Republic of Korea. Methods: The participants were 300 women residing in three rural regions. Data were collected from 1 August to 15 September 2025, in cooperation with the Lifestyle Improvement Association. Results: Descriptive and multiple regression analyses were conducted using the collected data. The prevalence of metabolic syndrome was 46.3%, and 36.7% of the participants had one or two risk factors. Factors that significantly influenced metabolic syndrome included obesity (β = 0.36, *p* < 0.001), health literacy (β = −0.25, *p* = 0.015), health responsibility (β = 0.25, *p* < 0.001), subjective health status (β = −0.25, *p* < 0.001), and menopause (β = 0.16, *p* = 0.005), which explained 36.3% of the variance (*F* = 8.59, *p* < 0.001). Conclusions: Enhancing health literacy and promoting a stronger sense of health responsibility may help reduce the prevalence of metabolic syndrome among middle-aged and older women in rural Republic of Korea.

## 1. Introduction

Metabolic syndrome (MetS) refers to the concurrent presence of three or more metabolic risk factors, including abdominal obesity, hypertension, hypertriglyceridemia, hyperglycemia, and low high-density lipoprotein (HDL) cholesterol levels, which substantially increase the risk of cardiovascular disease and diabetes [1]. Globally, the prevalence of MetS continues to rise [2], with reported rates ranging from 12.5% to 31.4%, depending on the population studied [3]. In Republic of Korea, approximately 24.9% of adults aged 19 years or older are affected, with substantially higher rates among older adults-particularly women aged 65 years and above (48.8%) compared to men (44.8%) [4]. The prevalence also rises significantly during middle age, particularly among those in their 40s, when the incidence of chronic diseases begins to increase [5].

MetS is higher in rural than urban populations, largely because of dietary factors such as excessive salt intake, higher obesity rates, and an aging demographic structure [6]. These trends indicate that middle-aged and older women living in rural areas are at high risk for developing MetS. This heightened vulnerability is often associated with hormonal changes after menopause that promote visceral fat accumulation, which is a major diagnostic component of MetS [7]. MetS more than doubles the risk of cardiovascular and cerebrovascular diseases [8] and increases the likelihood of diabetes, hypertension, stroke, kidney disease, and cancer, ultimately contributing to premature mortality [9]. Accordingly, identifying risk factors for MetS among rural women is critical for reducing the chronic disease burden and addressing health inequities between urban and rural communities.

The onset and progression of MetS are influenced not only by biological and lifestyle factors but also by psychosocial determinants such as health literacy [10,11]. Health literacy, defined as the ability to obtain, understand, and apply health-related information to make informed health decisions [12,13,14], has emerged as a critical factor in disease prevention and management. Higher health literacy is associated with improved self-care, healthier lifestyle behaviors, and lower risk of cardiovascular and metabolic diseases [15,16]. Conversely, individuals with low health literacy are more likely to have unhealthy behaviors, limited access to healthcare, and poorer health outcomes [17]. Low health literacy is particularly prevalent among older adults, those with lower education or income levels, and manual laborers, and it tends to be more pronounced in rural areas than in urban settings [18,19,20]. In particular, rural women exhibit higher rates of abdominal obesity, hyperglycemia, and hypertension, which are key indicators of MetS [21]. Therefore, examining the role of health literacy in the development and management of MetS in rural women is of growing importance.

Social support is a key psychosocial determinant of MetS [22]. Insufficient support from family, friends, or peers has been linked to poor health behaviors and worsening metabolic outcomes [23], whereas strong social networks have been shown to promote healthy lifestyle habits, such as regular physical activity and improved dietary practices [24]. Adequate social support can reduce central obesity, fasting blood glucose, and triglyceride levels, which are major components of MetS [25]. Moreover, peer-based encouragement and group participation are critical for sustaining long-term lifestyle modifications [23]. However, rural women often have limited healthcare access and smaller social networks, which contribute to lower levels of health-promoting behaviors and higher susceptibility to MetS [10,25].

MetS is often described as a “lifestyle disease,” highlighting the central role of behavioral modification in its prevention and management [2]. Unhealthy behaviors, such as physical inactivity, poor diet, smoking, alcohol consumption, stress, and obesity, are strongly associated with MetS development [26]. Although several studies have investigated individual lifestyle factors, most have analyzed them in isolation rather than comprehensively examining health-promoting behavioral patterns [10]. This study addresses this gap using reliable and valid measures to evaluate five core domains of health-promoting behaviors: health responsibility, physical activity, dietary habits, stress management, and smoking behavior.

This study investigated the effects of health literacy, social support, and health-promoting behaviors on MetS in middle-aged and older women living in rural Republic of Korea. By identifying modifiable psychosocial and behavioral factors, these findings can inform targeted intervention strategies that promote metabolic health and reduce chronic disease disparities in rural populations.

## 2. Materials and Methods

### 2.1. Study Design

This study used a descriptive survey design to examine the prevalence of MetS and identify the effects of health literacy, social support, and health-promoting behaviors on MetS among middle-aged and older women in rural areas.

### 2.2. Participants

The participants were middle-aged and older women residing in three rural regions of Republic of Korea, each with a population below 150,000 people. Convenience sampling was used to recruit participants aged 40 years or older who understood the study’s purpose, and voluntarily provided written informed consent. The required sample size was calculated using G*Power (version 3.1.9.7; Heinrich, Heine University Düsseldorf, Düsseldorf, Germany) for a two-tailed test with a significance level (α) of 0.05, statistical power (1−β) of 0.95, and medium effect size (d) of 0.15 for multiple regression. Considering 16 general and health-related characteristics and 9 key independent variables (including subfactors), the estimated minimum sample size was 242. Accounting for potential dropouts, 320 questionnaires were distributed. After excluding 20 incomplete or invalid responses, 300 participants were included in the final analysis.

### 2.3. Measures

A self-administered questionnaire was used to collect data on participants’ general and health-related characteristics, health literacy, social support, and health-promoting behaviors. Information on MetS was obtained from national health screening data collected from rural and urban residents in Republic of Korea. The following seven general characteristics were included: age, marital status, cohabitation with family, educational level, occupation, monthly income, and participation in social activities. Furthermore, nine health-related characteristics were assessed: accessibility to health clinics, media use, perceived health status, menopausal status, obesity, sleep duration, smoking, alcohol consumption, and exercise habits. Alcohol consumption was classified according to the Korea Disease Control and Prevention Agency on high-risk drinking guidelines, which define high-risk drinking in women as consuming five or more alcoholic drinks per occasion at least twice per week, or engaging in binge drinking (≥5 drinks in a single occasion) at least once per month [27]. All measurement tools included in this study were used with permission from their respective developers.

#### 2.3.1. Health Literacy

Health literacy was assessed using the European Health Literacy Survey Questionnaire (HLS-EU-Q47) adapted for use in the Korean population by Kim et al. [19]. The process involved forward–backward translation and linguistic validation to ensure cultural and contextual appropriateness for the Korean population.

The tool comprises 47 items across three health-related domains (healthcare, disease prevention, and health promotion) and four information-processing stages (accessing, understanding, appraising, and applying information). Each item is rated on a 4-point Likert scale ranging from 1 = very difficult to 4 = very easy, with an additional response option of “don’t know/not applicable” (excluded from analysis).

To calculate the Health Literacy Index, participants were required to respond to at least 43 of the 47 items, with minimum item thresholds for each subdomain (≥15 of 16 items for healthcare, ≥14 of 15 for disease prevention, and ≥14 of 16 for health promotion). The index was calculated using the following formula:

$$Index=\left(\frac{Mean-1}{3}\right)\times 50$$

where Mean represents the mean score of all valid responses, 1 is the minimum possible value, 3 is the range of the mean, and 50 is the maximum value of the new metric.

The scores were categorized as follows:

0–25: Inadequate health literacy

26–33: Problematic

34–42: Sufficient

43–50: Excellent

Cronbach’s α coefficients for the original HLS-EU-Q47 were 0.97 (general), 0.91 (healthcare), 0.91 (disease prevention), and 0.92 (health promotion). These values are identical to those reported by Kim et al. [19]. In this study, Cronbach’s α values were 0.96 (general), 0.92 (healthcare), 0.90 (disease prevention), and 0.91 (health promotion).

#### 2.3.2. Social Support

Social support was measured using the Medical Outcomes Study–Social Support Survey (MOS-SSS), originally developed by Sherbourne and Stewart [28] and translated into Korean by Lim [29]. The instrument comprises 19 items across four subscales: emotional/informational support (8 items), tangible support (3 items), affectionate support (3 items), and positive social interaction (3 items). Each item is rated on a 5-point Likert scale (1 = never to 5 = always), with higher scores indicating greater perceived social support. Cronbach’s α was 0.98 in the original study [28], 0.98 in Lim’s [29] validation, and 0.97 in this study.

#### 2.3.3. Health-Promoting Behaviors

Health-promoting behaviors were assessed using a modified version of the Health-Promoting Lifestyle Profile (HPLP) developed by Walker et al. [30] and adapted by Song et al. [31] for use with Korean adults. The instrument includes 25 items across five domains: health responsibility (5 items), physical activity (4 items), dietary habits (8 items), stress management (5 items), and smoking behavior (3 items). Each item is rated on a 4-point Likert scale (1 = never, 2 = sometimes, 3 = often, and 4 = always). Higher scores indicate greater engagement in health-promoting behaviors. Cronbach’s α was 0.92 in the original study [30], 0.80 in Song et al. [31], and 0.89 in this study. The subscale reliability coefficients were as follows: health responsibility (0.80), physical activity (0.80), dietary habits (0.81), stress management (0.72), and smoking behavior (0.62).

#### 2.3.4. Metabolic Syndrome

MetS was defined based on the Modified National Cholesterol Education Program Adult Treatment Panel III (NCEP ATP III) criteria and International Diabetes Federation (IDF) Asian guidelines for central obesity [32]. Anthropometric and biochemical data (waist circumference, blood pressure, fasting blood glucose, triglyceride levels, and HDL cholesterol levels) were obtained from participants’ physical measurements and blood test results, along with information on pharmacological treatment (antihypertensive, antidiabetic, and lipid-lowering medications). Based on previous studies [10,33,34], the number of diagnostic criteria for metabolic syndrome met by each participant was summed to calculate a MetS score. Participants were classified as follows: a score of 0 if none of the five criteria were met; 1 if one criterion was met; 2 if two criteria were met; 3 if three criteria were met; 4 if four criteria were met; and 5 if all five criteria were met. Higher scores indicated a greater risk of developing MetS.

### 2.4. Data Collection

This study was approved by the Institutional Review Board (IRB) of the authors’ university (IRB No. DGU IRB 20250021). Data were collected from 1 August to 15 September 2025. The researcher visited local Lifestyle Improvement Associations, which are community organizations dedicated to improving rural women’s welfare, in three cities. After explaining the study’s purpose and procedures, permission was obtained from the association’s chairpersons and staff to conduct the survey. Participants were informed verbally and in writing about the study’s purpose and procedures, voluntary participation, anonymity, right to withdraw, and potential benefits or risks. Written informed consent was obtained from all participants before data collection. Each participant received a sealed envelope containing the questionnaire, which was completed in approximately 20 min. Completed surveys were immediately collected, sealed, and stored securely. The participants were provided with a small token of appreciation upon completion. All data were digitized after collection, and both questionnaires and consent forms were stored in a locked cabinet and safely destroyed after the retention period to prevent breaches of personal information.

### 2.5. Data Analysis

Data were coded and analyzed using IBM SPSS version 29 (Armonk, NY, USA). Descriptive statistics, including frequencies, percentages, means, and standard deviations, were used to summarize general and health-related characteristics. Means and standard deviations were calculated for health literacy, social support, health-promoting behaviors, and MetS scores. Differences in MetS scores according to general characteristics were analyzed using *t*-tests and one-way ANOVA, followed by Scheffe’s post hoc tests. Pearson’s correlation coefficients were used to examine the relationships among health literacy, social support, health-promoting behaviors, and MetS. Factors influencing MetS were analyzed using multiple regression analysis.

## 3. Results

### 3.1. Participants’ General and Health-Related Characteristics

The mean participant age was 59.88 years, with 72% classified as middle-aged (40–64 years) and 28% as older (≥65 years). Most participants were married (85.7%) and lived with family members (86.3%). Regarding educational attainment, 48.7% had completed high school, whereas 25.6% had an educational level below middle school. Most participants were engaged in agricultural work (56.3%), followed by those who identified as housewives (13.7%). For average monthly household income, 39.7% of participants reported between 2.5 and 4.5 million KRW, 32% reported below 2.5 million KRW, and 28.3% reported above 4.5 million KRW. Slightly more than half (51.3%) did not participate in any social activities.

Regarding health-related characteristics, 51.3% reported easy access to health clinics or hospitals, 31.4% reported moderate access, and 17.3% reported difficulties. Most participants (68.0%) used media daily. Subjective health perception was “poor” in 33% of respondents and “good” in 16.7%. Most participants (85.7%) were postmenopausal, with a mean menopause age of 51.3 years. Approximately 30% were diagnosed with obesity. Their average sleep duration was 6.45 h, with 55% sleeping for less than seven hours per night. Most participants were nonsmokers (98%), while 45.3% reported no alcohol consumption, 39.7% reported low-risk drinking, and 15% reported high-risk drinking. Only 14.3% exercised regularly three or more times per week, whereas 43.7% reported irregular exercise (Table 1).

### 3.2. Health Literacy, Social Support, Health-Promoting Behaviors, and Metabolic Syndrome

The mean health literacy score was 26.66 ± 7.85, with subdomain scores of 25.27 ± 8.31 for healthcare, 29.00 ± 8.18 for disease prevention, and 26.66 ± 7.85 for health promotion. Overall health literacy levels were inadequate in 45.7% of the participants, problematic in 35.4%, sufficient in 13.9%, and excellent in 5%, indicating that the majority had inadequate or limited health literacy (Table 2).

The mean social support score was 3.46 ± 0.86, with subdomain means of 3.48 ± 0.84 for emotional/informational support, 3.23 ± 0.94 for tangible support, 3.61 ± 0.96 for affectionate support, and 3.58 ± 0.95 for positive social interaction. The mean score for health-promoting behaviors was 2.63 ± 0.45, with subdomain scores of 2.52 ± 0.61 for health responsibility, 2.19 ± 0.68 for exercise, 2.79 ± 0.57 for healthy diet, 2.40 ± 0.59 for stress management, and 3.35 ± 0.71 for smoking cessation. The mean MetS score was 2.27 ± 1.50. The prevalence of MetS (≥3 risk factors) was 46.3%, while 36.7% of the participants had one or two risk factors and 17% had none (Table 3).

### 3.3. Differences in Metabolic Syndrome According to General Characteristics

Table 4 summarizes the differences in MetS scores according to participants’ general and health-related characteristics. Significant differences were found according to age (t = −3.82, *p* < 0.001), cohabitation status (t = 2.04, *p* = 0.043), education level (t = 10.20, *p* < 0.001), occupation (t = 3.92, *p* = 0.021), and monthly income (t = 6.09, *p* = 0.003). Among health-related characteristics, statistically significant differences were observed for access to healthcare resources (t = 6.61, *p* = 0.002), media use (t = 7.53, *p* < 0.001), subjective health perception (t = 20.81, *p* < 0.001), menopausal status (t = −3.41, *p* < 0.001), obesity (t = −8.98, *p* < 0.001), and exercise frequency (t = 7.67, *p* < 0.001).

### 3.4. Correlations Among Health Literacy, Social Support, Health-Promoting Behaviors, and Metabolic Syndrome

The correlations between health literacy, social support, health-promoting behaviors, and MetS are presented in Table 5. Significant negative correlations were observed between MetS and the subdomains of health literacy: healthcare (r = −0.16, *p* = 0.009), disease prevention (r = −0.13, *p* = 0.025), and health promotion (r = −0.24, *p* < 0.001). MetS was also negatively correlated with social support (r = −0.21, *p* < 0.001). Among the subdomains of health-promoting behaviors, exercise (r = −0.21, *p* < 0.001), stress management (r = −0.12, *p* = 0.045), and smoking cessation (r = −0.15, *p* = 0.008) were negatively correlated with MetS, while healthy diet showed a weak, non-significant correlation (r = −0.09, *p* = 0.109).

### 3.5. Factors Influencing Metabolic Syndrome

The results for the factors affecting metabolic syndrome are presented in Table 6. Multicollinearity testing indicated no significant issues. Tolerance values ranged from 0.225 to 0.919 (≥0.1), and variance inflation factor values ranged from 1.089 to 4.439 (<10). The Durbin–Watson statistic was 1.824, indicating no autocorrelation of the residuals. Thus, all regression assumptions were satisfied, and the model was deemed appropriate. Multiple regression analysis was performed with the health literacy, social support, and health-promoting behavior subdomains, along with general characteristics that were significant in the bivariate analyses (age, cohabitation status, education, occupation, income, access to healthcare, media use, subjective health status, menopause, exercise frequency, and obesity). The results identified the following significant predictors of MetS: obesity (β = 0.36, *p* < 0.001), health-promotion health literacy (β = −0.25, *p* = 0.015), health responsibility (β = 0.25, *p* < 0.001), subjective health status (β = −0.25, *p* < 0.001), and menopause (β = 0.16, *p* = 0.005). These variables collectively explained 36.3% of the variance in MetS scores (F = 8.59, *p* < 0.001).

Additionally, this study noted that although social support showed a significant negative correlation with MetS, it was not significant in the multivariable model. Conversely, certain subscales of health-promoting behavior were not significantly correlated with MetS but emerged as significant predictors in the multivariable analysis. To explore the possibility that health-promoting behavior mediates the relationship between social support and MetS, a mediation analysis was conducted using Baron and Kenny’s three-step regression procedure [35]. In the first step, social support significantly influenced all subcomponents of health-promoting behavior (responsibility: β = 0.36, *p* < 0.001; exercise: β = 0.32, *p* < 0.001; healthy eating: β = 0.27, *p* < 0.001; stress management: β = 0.51, *p* < 0.001; smoking cessation: β = 0.25, *p* < 0.001). In the second step, social support had a significant negative effect on MetS (β = −0.21, *p* < 0.001). In the third step, when both social support and the subcomponents of health-promoting behavior were entered simultaneously, social support remained a significant predictor of MetS (β = −0.19, *p* < 0.001), though the regression coefficient decreased compared to when it was entered alone. Among the subcomponents of health-promoting behavior, responsibility (β = 0.24, *p* < 0.001) and exercise (β = −0.21, *p* = 0.002) were significant predictors, whereas healthy eating (β = −0.04, *p* = 0.577), stress management (β = −0.01, *p* = 0.914), and smoking cessation (β = −0.11, *p* = 0.066) were not significant.

## 4. Discussion

This study examined the prevalence of MetS and the related effects of health literacy, social support, and health-promoting behaviors among middle-aged and older women residing in rural Republic of Korea. The prevalence of MetS in this study was 46.3%, with an additional 36.7% of the participants exhibiting one or two MetS risk factors. These rates are substantially higher than the national prevalence of 24.9% among Korean adults [4] and comparable to the 48.8% reported among women aged ≥65 years [4]. The prevalence observed in this study also exceeds the global range of 12.5–31.4% [3] and estimated 11.9–37.1% reported for the Asia Pacific region [35]. According to a study analyzing trends in MetS prevalence over 10 years in Korea (2007–2018), the increase in prevalence was more pronounced in rural areas compared to urban regions, with rates ranging from 30.4% to 34.7% in areas with a high proportion of rural residents [36]. Furthermore, the prevalence observed in the present study exceeds the 39.8% reported among rural adults aged ≥40 years by Lee et al. [5]. Among women, prevalence consistently increased with age, reaching 48.8% in those aged ≥65 years—higher than that observed in men of the same age group (44.8%) [4]. Taken together, previous research indicates that individuals living in rural areas are more susceptible to metabolic syndrome than urban residents, with particularly high prevalence among older women. Accordingly, the target population of this study—older women residing in rural regions—represents a group in which multiple social risk factors for metabolic syndrome converge. This finding supports the importance of addressing social determinants in the development of strategies for the prevention and management of metabolic syndrome.

These findings reaffirm the physiological mechanisms linking menopause, aging, and MetS. Declining estrogen levels after menopause contribute to increased visceral fat deposition and adverse lipid metabolism [37,38]. More than half of the participants in this study had a waist circumference ≥ 80 cm, indicating central obesity. These findings are consistent with previous reports that postmenopausal women experience substantial metabolic and body composition changes, leading to a 32–58% prevalence of MetS, which is significantly higher than that in premenopausal women [39]. Therefore, menopausal status appears to play a critical role in the risk of developing MetS in rural women. However, in this study, menopausal status was assessed using a single binary variable (“menopause: yes/no”), which did not account for important physiological factors such as menopausal transition stages, duration since menopause, or the use of hormone replacement therapy. This constitutes a limitation of the study. Future research should consider incorporating menopausal stage and relevant endocrine markers to more precisely investigate how menopausal transition affects the risk of MetS among middle-aged women living in rural areas.

The prevalence of MetS varied by age, socioeconomic status, media use, and health-related characteristics. Aging is a well-established risk factor for MetS [2], and participants aged ≥ 65 years showed a significantly higher prevalence. Likewise, lower educational attainment, limited income, and agricultural occupations, which are common characteristics among rural residents, were associated with a greater risk of MetS. These findings support those of previous studies showing that low socioeconomic status increases susceptibility to MetS [5,40]. High-calorie, carbohydrate-rich, and sodium-heavy diets are more common in rural areas because they are inexpensive and accessible [5]. Physical inactivity further increases this risk [38]. Rural middle-aged and older women often prioritize agricultural labor over structured exercise, resulting in reduced engagement in health-promoting activities [41]. Rural residents generally report less frequent physical activity than urban residents [5]. Furthermore, participants who reported poor access to healthcare facilities and limited media use exhibited a higher risk of MetS, emphasizing the role of healthcare accessibility and exposure to health information in disease prevention.

The participants had a mean health literacy score of 24.7, indicating generally inadequate health literacy, with 81.1% categorized as inadequate or problematic. This score was substantially lower than the national average of 34.5 among Korean adults [19], as well as those reported in Europe (33.8) and Taiwan (34.4). These results reflect the interplay between rural residence and sociodemographic characteristics, such as advanced age, low education, and limited income. Similarly, Kim et al. [19] found lower levels of health literacy among individuals who were older, less educated, or engaged in manual labor. Chun and Lee [20] also reported lower health literacy among rural residents than among urban residents, supporting the present findings.

Notably, although 68% of the participants reported daily media use, their health literacy remained low. This indicates that the challenge lies not only in accessing information but also in comprehending, evaluating, and applying it effectively. According to Park et al. [42], the reliability and interpretability of online health information may pose particular difficulties for rural populations. Moreover, digital literacy (i.e., the ability to use smartphones and Internet-based platforms effectively) has been positively correlated with health literacy [43]. Therefore, disparities in digital competence may exacerbate health literacy gaps between rural and urban populations. Thus, health education programs targeting rural women should emphasize not only content delivery but also digital health literacy training to enhance comprehension and practical application of health information.

Among the variables examined, obesity, perceived health status, menopause, health-promotion-related health literacy, and health responsibility were identified as significant predictors of MetS. The strong association between obesity and MetS aligns with prior findings indicating that hormonal changes and reduced physical activity following menopause contribute to visceral fat accumulation and metabolic disturbances [38,44]. These physiological changes result in central obesity, which is a hallmark of MetS. Previous studies have demonstrated that increased waist circumference after menopause is a critical determinant of MetS, whereas higher physical activity levels have a protective effect [45]. Although this study did not directly measure activity intensity, previous research has indicated that middle-aged and older Korean women primarily engage in low-intensity walking and rarely meet recommended aerobic or strength exercise levels [46]. This evidence supports the hypothesis that hormonal and behavioral factors jointly increase the risk of MetS in postmenopausal women.

Perceived health status was also a significant determinant of MetS. Participants with poorer self-rated health also had a higher risk of developing MetS. These findings are consistent with those of prior research showing that individuals who perceive their health positively are more likely to engage in health-promoting behaviors [46] and exhibit a lower prevalence of MetS [47]. Thus, subjective health perception may be a behavioral and psychological indicator of metabolic health.

Lower health literacy was significantly associated with a higher risk of MetS, corroborating previous findings that women with higher health literacy experience fewer dyslipidemia-related cardiovascular complications [18]. Similarly, studies among men have shown that higher health literacy is linked to healthier lifestyles and reduced MetS prevalence [11]. Interventions aimed at improving health literacy have been shown to enhance health behaviors and prevent chronic disease progression [46]. Health literacy deficits are more common among older, low-income, and less-educated populations [19]; therefore, tailored interventions to strengthen health literacy are essential to mitigate the risk of MetS in rural communities. The World Health Organization [48] emphasizes that improving health literacy requires national leadership, sustained funding, and integrated education, policy, and evaluation frameworks. For example, the United States has implemented a National Action Plan to promote accessible health information and community-based education [49]. In contrast, only approximately 60% of Korean adults demonstrate adequate health literacy, with marked disparities in age, education, and socioeconomic status, underscoring the need for targeted interventions for vulnerable populations [50].

Unexpectedly, the health responsibility subscale of health-promoting behaviors showed no linear association with MetS in the bivariate analysis but emerged as one of the strongest predictors in the multivariable model. Previous studies have characterized health responsibility behaviors, such as undergoing health checkups, consulting healthcare professionals, and seeking health information, as preventive in nature [47]. However, other studies have indicated that these behaviors often increase after disease onset as a compensatory response [51]. Considering that 83% of the participants in this study had at least one MetS risk factor and were taking medications such as antihypertensives (35.0%), antidiabetic agents (19.7%), and lipid-lowering drugs (45.3%), and that the majority were postmenopausal, their elevated health responsibility may have reflected post-diagnosis management behaviors rather than proactive prevention. Thus, future longitudinal studies should be conducted to examine the temporal progression of health responsibility behaviors across the disease continuum.

Among the subcomponents of health-promoting behavior, exercise, healthy eating, stress management, and smoking cessation showed significant associations with MetS in the bivariate analysis but were not significant predictors in the multivariable model. Contrary to our findings, previous studies have reported that healthy lifestyle practices can reduce metabolic syndrome risk. In particular, regular moderate-intensity exercise performed for at least 30 min daily has been shown to increase muscle mass and stimulate fatty acid oxidation, resulting in improved lipid profiles, reduced blood pressure, and decreased insulin resistance, thereby lowering the risk of MetS [52]. A comparative study of lifestyle patterns between rural and urban populations also reported that rural residents engage in regular physical activity less frequently and consume higher amounts of carbohydrates and sodium compared to urban residents [5].

Although social support did not have a direct effect on MetS in the multivariable model in the present study, it demonstrated an indirect effect mediated by health-promoting behaviors—specifically, responsibility and exercise. This finding suggests that social support may not directly reduce the incidence of MetS but may instead function as a process-oriented factor that motivates lifestyle modification and promotes sustained engagement in healthy behaviors. Previous research has also identified social support as a protective factor that facilitates healthy lifestyle adherence and reduces the risk of MetS development [10]. Based on this evidence, social support may have limited observable effects when included as an isolated variable; however, it may act as a latent factor that influences MetS through its role in facilitating behavior change. Therefore, future research should examine the causal pathways among social support, health-promoting behaviors, and MetS using structural equation modeling (SEM) to examine the underlying mechanisms through which social support may reduce MetS risk in rural women.

Collectively, these findings have several key implications. First, physiological factors, such as menopause and obesity, along with psychosocial factors, including health literacy and perceived health, play a central role in the development of MetS among rural women. Second, health literacy is not only an indicator of information access but also a determinant of behavioral competence and self-management capacity. Finally, this study underscores the importance of tailored health education and health information delivery systems that address the contextual reality of rural populations. Therefore, strengthening community-based health literacy initiatives and integrating digital health training may serve as effective strategies to reduce the risk of MetS and promote health equity in this vulnerable population.

## 5. Limitations

This study has several limitations that should be considered when interpreting the findings. First, the participants were selected through convenience sampling from three small rural regions in Korea, which may have limited the representativeness of the sample. This approach was chosen due to practical constraints in accessing the specific target population of older women residing in rural areas, taking into account the feasibility of data collection and study implementation. However, the use of convenience sampling presents limitations, including reduced representativeness of the sample and restricted generalizability of the study findings. Therefore, generalization of the results to all rural women should be approached with caution. Second, this study used a cross-sectional design, thereby preventing causal relationships between health literacy, social support, health-promoting behaviors, and MetS from being definitively established. Future longitudinal studies are required to examine the causal pathways and temporal relationships among these variables. Third, the primary variables were assessed using self-report questionnaires, which may have introduced recall bias and subjective judgment. Factors such as obesity, health behaviors, and health literacy were not cross-validated with objective clinical or biomedical data, potentially affecting measurement accuracy. Fourth, although MetS was defined based on health examination results, other relevant clinical factors, including medication use, healthcare utilization, and physical activity levels, were not controlled for comprehensively. Therefore, the potential influence of residual confounding variables cannot be entirely excluded. Finally, the study participants were primarily postmenopausal women with a relatively high mean age, restricting the applicability of the findings to younger or male populations. Despite these limitations, this study provides meaningful empirical evidence on health literacy, social support, and health-promoting behaviors associated with MetS among middle-aged and older rural women. These findings can provide a valuable foundation for future intervention studies and community-based health-promotion strategies aimed at this population.

## 6. Conclusions

This study investigated the effects of health literacy, social support, and health-promoting behaviors on the prevalence of MetS in middle-aged and older women living in rural areas. The findings identified obesity, health-promotion-related health literacy, health responsibility, perceived health status, and menopausal status as significant predictors of MetS risk. Higher levels of health literacy were associated with a lower risk of MetS, suggesting that individuals who better understand and utilize health information are more likely to adopt preventive behaviors and maintain their metabolic health. Conversely, participants with obesity, poor perceived health, or menopausal status exhibited greater MetS risk, which is consistent with prior research. These results highlight the importance of enhancing health literacy and fostering active health responsibility behaviors to mitigate metabolic risks among rural women. Public health interventions should move beyond individual behavioral changes and incorporate community-based approaches that improve access to reliable health information and healthcare services. Tailored education and counseling programs that reflect the specific needs, socioeconomic conditions, and digital literacy of rural women may enhance health-promoting behaviors, reduce health disparities, and ultimately improve the quality of life of this vulnerable population.

## Figures and Tables

**Table 1:** Participant characteristics (N = 300).

Characteristics	Categories	*n* (%)	M ± SD
General characteristics			
Age (year)	40~65	216 (72.0)	59.88 ± 8.15
	>65	84 (28.0)	
Marital status	Married	257 (85.7)	
	Others	43 (14.3)	
Living with	Family	259 (86.3)	
	Alone	41 (13.7)	
Education level	Elementary school	37 (12.3)	
	Middle school	40 (13.3)	
	High school	146 (48.7)	
	College or above	77 (25.7)	
Occupation	Housewife	41 (13.7)	
	Farming	169 (56.3)	
	Others	90 (30.0)	
Monthly Income (Won)	<2,500,000	96 (32.0)	
	2,500,000~4,500,000	119 (39.7)	
	>4,500,000	85 (28.3)	
Community participation	Yes	146 (48.7)	
	No	154 (51.3)	
Health-related characteristics			
Access of health clinic	Not useful	52 (17.3)	
	Moderate	94 (31.4)	
	Useful	154 (51.3)	
Use of media	Rarely use	34 (11.3)	
	Several times per week	62 (20.7)	
	Daily use	204 (68.0)	
Perceived health	Bad	99 (33.0)	
	Moderate	151 (50.3)	
	Good	50 (16.7)	
Menopause	Yes	257 (85.7)	51.32 ± 3.10
	No	43 (14.3)	
Obesity	Yes	90 (30.0)	
	No	210 (70.0)	
Sleeping time	1~7	165 (55.0)	6.45 ± 1.26
	>7	135 (45.0)	
Smoke	None	294 (98.0)	
	Yes	6 (2.0)	
Alcohol	None	136 (45.3)	
	Low-risk (≤4 drinks/week)	119 (39.7)	
	High-risk (>4 drinks/week)	45 (15.0)	
Exercise	None	126 (42.0)	
	Irregular (<3/week)	131 (43.7)	
	Regular	43 (14.3)	

M = mean; SD = standard deviation.

**Table 2:** Mean health literacy scores and percentage of each health literacy level category (N = 300).

Health Literacy	Total (M ± SD)	Level *			
		Inadequate *n* (%)	Problematic *n* (%)	Sufficient *n* (%)	Excellent *n* (%)
General health literacy index (*n* = 280)	26.66 ± 7.85	128 (45.7)	99 (35.4)	39 (13.9)	14 (5.0)
Healthcare literacy index (*n* = 277)	25.27 ± 8.31	147 (53.1)	87 (31.4)	30 (10.8)	13 (4.7)
Disease prevention literacy index (*n* = 283)	29.00 ± 8.18	91 (32.2)	113 (39.9)	58 (20.5)	21 (7.4)
Health promotion literacy index (*n* = 291)	26.66 ± 7.85	157 (54.0)	76 (26.1)	45 (15.5)	12 (4.5)

* 0–25 points, inadequate; >25–33 points, problematic; >33–42 points, sufficient; >42–50 points, excellent.

**Table 3:** Social support, health promotion behaviors, and metabolic syndrome (N = 300).

Variables	Categories	*n* (%) or M ± SD	Min, Max	Range
Social support	Total	3.46 ± 0.86	1.11, 5.00	1–5
	Emotional/informational	3.48 ± 0.84	1.25, 5.00	1–5
	Tangible	3.23 ± 0.94	1.00, 5.00	1–5
	Affectionate	3.61 ± 0.96	1.00, 5.00	1–5
	Positive social interaction	3.58 ± 0.95	1.00, 5.00	1–5
Health promoting	Total	2.63 ± 0.45	1.24, 3.72	1–4
behaviors	Health responsibility	2.52 ± 0.61	1.00, 4.00	1–4
	Exercise	2.19 ± 0.68	1.00, 4.00	1–4
	Healthy diet	2.79 ± 0.57	1.25, 4.00	1–4
	Stress management	2.40 ± 0.59	1.00, 3.80	1–4
	Smoking cessation	3.35 ± 0.71	1.00, 4.00	1–4
Metabolic	Total	2.27 ± 1.50	0.00, 5.00	0–5
	None	51 (17.0)		
syndrome	Risk group (1~2)	110 (36.7)		
	Patient (>3)	139 (46.3)		
	Abdominal obesity *	169 (56.3), 83.56 ± 9.48	62, 120	
	High blood pressure **	138 (46.0)		
	Systolic BP	124.41 ± 12.06	100, 165	
	Diastolic BP	77.02 ± 8.45	42, 96	
	On medication for hypertension	105 (35.0)		
	Impaired fasting glucose ***	110 (36.7), 99.03 ± 18.27	67, 261	
	On medication for diabetes	59 (19.7)		
	Hypertriglyceridemia ****	170 (56.7), 134.95 ± 58.96	38, 494	
	On medication for hyperlipidemia	136 (45.3)		
	Low high-density lipoprotein-cholesterol *****	93 (31.0), 58.71 ± 14.15	21, 105	

Note: M = mean; SD = standard deviation; Min = minimum; Max = maximum; * waist circumference ≥ 80 cm; ** systolic BP ≥ 130 mmHg or diastolic BP ≥ 85 mmHg; *** ≥100 mg/dL; **** ≥150 mg/dL; ***** ≤50 mg/dL.

**Table 4:** Differences in metabolic syndrome by participant characteristics (N = 300).

Characteristics	Categories	M ± SD	t or F (*p*)
General characteristics			
Age (year)	40~65	2.06 ± 1.49	−3.82 (<0.001)
	>65	2.79 ± 1.40	
Marital status	Married	2.22 ± 1.46	1.27 (0.206)
	Others	2.53 ± 1.68	
Living with	Family	2.20 ± 1.47	2.04 (0.043)
	Alone	2.71 ± 1.62	
Education level	Elementary school ^a^	3.11 ± 1.43	10.20 (<0.001)
	Middle school ^b^	2.88 ± 1.32	
	High school ^c^	2.16 ± 1.51	
	College or above ^d^	1.75 ± 1.34	
	Scheffe		a > c, d; b > d
Occupation	Housewife ^a^	2.44 ± 1.57	3.92 (0.021)
	Farming ^b^	2.42 ± 1.50	
	Others ^c^	1.90 ± 1.41	
	Scheffe		b > c
Monthly Income (Won)	<2,500,000 ^a^	2.63 ± 1.47	6.09 (0.003)
	2,500,000~4,500,000 ^b^	2.27 ± 1.51	
	>4,500,000 ^c^	1.86 ± 1.42	
	Scheffe		a > c
Community participation	Yes	2.12 ± 1.51	−1.69 (0.091)
	No	2.41 ± 1.48	
Health-related characteristics			
	Not useful ^a^	2.90 ± 1.52	6.61 (0.002)
Access of health clinic	Moderate ^b^	2.28 ± 1.61	
	Useful ^c^	2.05 ± 1.36	
	Scheffe		a > b, c
Use of media	Rarely use ^a^	3.15 ± 1.54	7.53 (<0.001)
	Several times per week ^b^	2.34 ± 1.45	
	Daily use ^c^	2.10 ± 1.46	
	Scheffe		a > b, c
Perceived health	Bad ^a^	2.90 ± 1.46	20.81 (<0.001)
	Moderate ^b^	2.15 ± 1.42	
	Good ^c^	1.36 ± 1.26	
	Scheffe		a > b, c; b > c
Menopause	Yes	2.39 ± 1.46	−3.41 (<0.001)
	No	1.56 ± 1.58	
Obesity	Yes	3.32 ± 1.09	−8.98 (<0.001)
	No	1.81 ± 1.42	
Sleeping time	1~7	2.21 ± 1.53	−0.77 (0.440)
	>7	2.34 ± 1.47	
Smoke	None	2.26 ± 1.50	−0.66 (0.510)
	Yes	2.67 ± 1.75	
Alcohol	None	2.26 ± 1.55	0.03 (0.975)
	Low-risk (≤4 drinks/week)	2.25 ± 1.42	
	High-risk (>4 drinks/week)	2.31 ± 1.58	
Frequency of exercise	None ^a^	2.50 ± 1.50	7.67 (<0.001)
	Irregular ^b^ (<3/week)	2.30 ± 1.48	
	Regular ^c^	1.49 ± 1.33	
	Scheffe		a > c, b > c

M = mean; SD = standard deviation. a, b, c, d = superscripts denote statistical groupings based on the Scheffe post hoc analysis.

**Table 5:** Correlations among health literacy, social support, health-promoting behaviors, and metabolic syndrome (N = 300).

Variables	Categories	HL			SS	HPB					MetS
		Healthcare	Disease Prevention	Health Promotion		Health Responsibility	Exercise	Healthy Diet	Stress Management	Smoking Cessation	
		r (*p*)	r (*p*)	r (*p*)	r (*p*)	r (*p*)	r (*p*)	r (*p*)	r (*p*)	r (*p*)	r (*p*)
HL	Healthcare	1									
	Disease prevention	0.79 (<0.001)	1								
	Health promotion	0.77 (<0.001)	0.81 (<0.001)	1							
SS		0.48 (<0.001)	0.45 (<0.001)	0.53 (<0.001)	1						
HPB	Health responsibility	0.39 (<0.001)	0.44 (<0.001)	0.53 (<0.001)	0.36 (<0.001)	1					
	Exercise	0.32 (<0.001)	0.33 (<0.001)	0.41 (<0.001)	0.32 (<0.001)	0.42 (<0.001)	1				
	Healthy diet	0.18 (0.003)	0.18 (0.002)	0.29 (<0.001)	0.27 (<0.001)	0.49 (<0.001)	0.40 (<0.001)	1			
	Stress management	0.36 (<0.001)	0.34 (<0.001)	0.48 (<0.001)	0.51 (<0.001)	0.51 (<0.001)	0.41 (<0.001)	0.52 (<0.001)	1		
	Smoking cessation	0.23 (<0.001)	0.29 (<0.001)	0.27 (<0.001)	0.25 (<0.001)	0.27 (<0.001)	0.22 (<0.001)	0.30 (<0.001)	0.27 (<0.001)	1	
MetS		−0.16 (0.009)	−0.13 (0.025)	−0.24 (<0.001)	−0.21 (<0.001)	0.03 (0.597)	−0.21 (<0.001)	−0.09 (0.109)	−0.12 (0.045)	−0.15 (0.008)	1

HL, health literacy; SS, social support; HPB, health-promoting behaviors; MetS, metabolic syndrome.

**Table 6:** Factors influencing metabolic syndrome among participants (N = 300).

Variables	B	SE	ß	t (*p*)	95% CI Min, Max
(Constant)	1.63	0.65		2.50 (0.013)	0.35, 2.91
Age (reference ≤ 65 years)	0.27	0.20	0.08	1.40 (0.164)	−0.11, 0.66
Living with (reference = alone)	0.07	0.23	0.02	0.28 (0.781)	−0.40, 0.53
Education (reference = elementary school)	−0.22	0.11	−0.13	−1.92 (0.056)	−0.43, 0.01
Occupation (reference = housewife)	0.01	0.13	0.01	0.05 (0.964)	−0.25, 0.26
Monthly income (reference ≤ 2,500,000 won)	0.11	0.12	0.06	0.87 (0.388)	−0.14, 0.35
Access of source (reference = not use)	−0.09	0.11	−0.04	−0.80 (0.423)	−0.30, 0.13
Use of media (reference = not use)	−0.12	0.13	−0.05	−0.95 (0.342)	−0.37, 0.13
Perceived health (reference = bad)	−0.52	0.11	−0.25	−4.64 (<0.001)	−0.75, −0.30
Menopause (reference = none)	0.68	0.24	0.16	2.85 (0.005)	0.21, 1.15
Frequency of exercise (reference = no)	−0.19	0.13	−0.09	−1.46 (0.145)	−0.45, 0.07
Obesity (reference = no)	1.19	0.17	0.36	7.09 (<0.001)	0.86, 1.52
Healthcare HL	0.01	0.02	0.08	0.88 (0.379)	−0.02, 0.05
Disease promotion HL	0.02	0.02	0.13	1.39 (0.167)	−0.01, 0.06
Health promotion HL	−0.04	0.02	−0.25	−2.44 (0.015)	−0.07, −0.01
Social support	0.11	0.12	0.06	0.94 (0.347)	−0.12, 0.35
Responsibility HPB	0.59	0.16	0.25	3.70 (<0.001)	0.27, 0.90
Exercise HPB	−0.06	0.14	−0.03	−0.40 (0.693)	−0.34, 0.23
Healthy eat HPB	−0.26	0.17	−0.10	−1.56 (0.121)	−0.59, 0.07
Stress management HPB	−0.04	0.18	−0.02	−0.24 (0.813)	−0.39, 0.31
Smoking cessation HPB	−0.06	0.11	−0.03	−0.49 (0.624)	−0.28, 0.17
	R_2_ = 0.410 Adj. R_2_ = 0.363, F = 8.593, *p* < 0.001				

HL = health literacy; HPB = health-promoting behaviors; Min = minimum; Max = maximum; CI = confidence interval.

## Data Availability

Data are available upon request due to ethical and privacy restrictions.
